# Trajectories of generalized anxiety disorder, major depression and change in quality of life in adults aged 50 + : findings from a longitudinal analysis using representative, population-based data from Ireland

**DOI:** 10.1007/s00127-022-02373-0

**Published:** 2022-10-13

**Authors:** Johanna Katharina Hohls, Hans-Helmut König, André Hajek

**Affiliations:** Department of Health Economics and Health Services Research, University Medical Center Hamburg-Eppendorf, Martinistr. 52, 20246 Hamburg, Germany

**Keywords:** Generalized anxiety disorder, Major depression, Quality of life, Older adults, Longitudinal study, The Irish Longitudinal Study on Aging (TILDA)

## Abstract

**Purpose:**

To investigate the longitudinal association between trajectories (incidence, remission) of generalized anxiety disorder (GAD), major depression (MD) and change in quality of life (QoL) in adults aged 50 + , and to assess the symmetry in these relationships using observational study data.

**Methods:**

Data were derived from two waves of The Irish Longitudinal Study on Aging (2014–2015, wave 3: *n* = 6400; 2016, wave 4: *n* = 5715), a nationally representative cohort of community-dwelling adults aged 50 +. GAD and MD were assessed by means of the short form of the Composite International Diagnostic Interview. QoL outcomes were assessed using the Control, Autonomy, Self-realization, and Pleasure scale (CASP-12 with two domains *control/autonomy and self-realization/pleasure*). Covariate-adjusted, asymmetric fixed effects panel regressions and post-estimation Wald tests were used for statistical analysis.

**Results:**

Regarding incident disorders, only incident MD was significantly associated with a reduction in QoL over time (*control/autonomy* domain: *b* = − 0.74, SE: 0.30). Regarding remission, both remission of MD (*b* = 0.61, SE: 0.20) and remission of GAD (*b* = 0.61, 0.26) were significantly associated with an increase in the *self-realization/pleasure* domain over time. Subsequent Wald tests of the estimates were not significant, indicating symmetric effects.

**Conclusion:**

Particularly the remission of GAD and MD was associated with a significant improvement in one of the QoL domains, indicating domain- and trajectory-specific differences. However, symmetric effects observed in this study indicate that gains and losses in QoL associated with remission and incidence of GAD and MD are of similar magnitude in adults aged 50 +.

**Supplementary Information:**

The online version contains supplementary material available at 10.1007/s00127-022-02373-0.

## Introduction

Generalized anxiety disorder (GAD) and major depression (MD) are common mental disorders in older adults from the general population [1, 2], with estimates suggesting a prevalence rate of 3.3% for current MD (lifetime prevalence: 16.5%) and of 2.3% for current GAD (lifetime prevalence: 6.4%) in people aged 50 and over [3]. The analysis of mental health and its effects in older age is highly relevant, as previous research shows a poor course, such as high relapse rates and chronicity, in older adults suffering from depression or anxiety [4–6]. An increased economic burden has also been associated with these disorders [7, 8]. In addition, numerous previous studies have analyzed the association between GAD, MD and quality of life (QoL) [9–13]. Among these, several cross-sectional studies have found that the presence of these disorders was generally related to lower QoL, the association was not always uniform across all QoL domains analyzed [9, 10, 13]. Two longitudinal studies on the same general population sample only focused on one health-related QoL domain and found an increase in QoL associated with new onset of GAD or MD [11] as well as a reduction in QoL associated with remission of these disorders [12]. However, most studies on this association have been either of cross-sectional nature, conducted in mental health samples recruited in healthcare settings, or in mixed-age samples. The relationship between GAD, MD and QoL has rarely been investigated in longitudinal, observational studies of adults aged 50 + from the general population. Yet, due to demographic ageing, focusing on the association between mental health and QoL in older adults is highly relevant. Moreover, mental health as well as QoL are of great importance for successful ageing [14, 15].

The use of longitudinal data has several advantages over cross-sectional data. For example, intra-individual trajectories of symptoms and factors associated with these trajectories can be analyzed [16]. Consequently, the effect of incidence or remission of a disorder on change in QoL over time can be evaluated in the same sample. Some studies have investigated this using data from large, population-based observational cohorts of adults of mixed-age groups. For example, based on data from the National Epidemiologic Survey on Alcohol and Related Conditions, it was reported that incident MD and GAD were associated with the largest decrements in QoL over time compared to other psychiatric disorders [11], and that remission of these disorders was associated with a significant improvement in QoL in participants aged 18 and over [12]. However, the authors did not compare whether gains and losses in QoL associated with remission and incidence of the disorders differed significantly. Using data from the Netherlands Mental Health Survey and Incidence Study and its expansion, several studies have compared QoL scores in people between 18 and 64 years of age before and after an episode of MD and anxiety disorders [17–19]. For example, regarding MD, Buist-Bouwman et al. [19] found that, while QoL was mostly reduced during an episode of MD, the pre- and post-MD QoL levels did not differ significantly on most domains. This is indicative of a symmetric relationship, meaning that losses in QoL associated with the incidence of the mental disorder were likely of similar magnitude as QoL gains associated with the remission of the disorder. Only for mental health-related domains of the questionnaire did they find that mean post-MD QoL was significantly higher than pre-MD QoL within the cohort [19]. This indicates an asymmetric association in this QoL domain, i.e. gains in QoL associated with remission were presumably larger than the losses associated with the incidence of the disorder. While Bos et al. [17] found no significant difference between pre- and post-MD QoL levels in adjusted models, they extended previous findings by additionally examining whether these effects differed by type of disorder or severity of the episode, and found no significant differences. Investigating anxiety disorders, Schopman et al. [18] found similar results. Only for those with a recurrent anxiety disorder, they found a so-called “scarring effect” for mental health-related QoL, i.e. a lower level of QoL post-remission compared to pre-disorder levels, hinting at an asymmetric association. However, as results were not presented separately for individual anxiety disorders in this study, evidence for GAD in this respect is still scarce.

Some studies based on longitudinal, population-based cohort studies of adults aged 50 and over [English Longitudinal Study of Ageing (ELSA), The Irish Longitudinal Study on Ageing (TILDA)] have investigated the association between depression or depressive symptoms and QoL in people aged 50 + [20–23]. These studies applied a QoL measure specifically developed for application in older age and reflecting positive aspects of ageing (Control, Autonomy, Self-realization, and Pleasure scale, CASP [24]). While the negative association between depression measures and overall QoL (CASP total score) observed in the studies of adults aged 50 + are comparable to results observed in mixed-age samples described above, these studies did not analyze different CASP subdomains as outcomes, nor did they analyze the role of anxiety or GAD. As that anxiety and depression can be associated with QoL domains differently as shown and discussed in a recent systematic review and meta-analysis [25], focusing on CASP subdomains may build upon the existing studies and allow for more detailed and nuanced statements regarding this complex association.

In sum, while there is some evidence on the association between MD, GAD and QoL in general population samples as described above, there are some gaps in the current literature: Most studies have focused on MD or depressive symptoms rather than on GAD or anxiety symptoms. Moreover, evidence on the association between disorder-specific trajectories and change in QoL subdomains are lacking in community-dwelling adults aged 50 + . Lastly, there is little information on whether a symmetric or asymmetric association may be present in this relationship. Knowledge on whether there are domain- or disorder-specific asymmetric associations could be beneficial, as it may indicate areas where additional support or interventions in this age group may be needed.

Thus, in light of the previous research, the aim of this study was twofold: first, to analyze the association between specific changes in diagnostic status of GAD as well as MD (incidence, remission), and change in QoL domains over time in adults aged 50 + using longitudinal data from a large, population-based cohort study; and second, to assess the symmetry in this association.

## Methods

### The TILDA study

This is a secondary analysis of data from TILDA, a nationally representative sample of community-dwelling adults aged 50 years and over, residing in Ireland. The multi-disciplinary TILDA study collects information on a variety of aspects in this age group including health, economic and social circumstances. The design, sampling procedure, and participant flow of the TILDA study have been described in detail previously [26–28]. In short, a random sample was selected using a multi-stage sampling procedure [RANSAM; 29]. At first, residential addresses in the Republic of Ireland were grouped into first-stage clusters. Second, out of these clusters, 640 were selected due to proportionate stratification by socioeconomic status, age, and geographical location. Clusters for inclusion were selected randomly, with the probability of selection proportional to the estimated number of people aged 50 and over in each cluster. Finally, a randomly selected set of 40 addresses in each cluster was selected for immediate issue. In the selected households, all persons aged 50 and over, as well as their spouses of any age, were asked to participate in the TILDA study. The response rate of all estimated eligible households (*n* = 10,128) was 62% (*n* = 6279). Written informed consent was obtained from all participants included in the study. The TILDA study was approved by the Trinity College Dublin Research Ethics Committee.

Data collection for wave 1 was conducted between October 2009 and February 2011. Follow-up assessments were conducted every 2 years thereafter (wave 2: February 2012–March 2013; wave 3: March 2014–October 2015; wave 4: January–December 2016). Each participant underwent a computer assisted home interview (administered by trained interviewers), was asked to return a paper-based self-completion questionnaire, and was invited to undertake an additional health assessment (at waves 1 and 3 only) [28]. The survey instrument has been tested and refined in two pilot studies [26].

For the purpose of this analysis, data from the most recent two waves of the TILDA study were used (waves 3 and 4, hereafter referred to as t_1_ and t_2_) [30, 31]. At these waves, as all variables of interest were available in the form needed for analysis in the dataset provided for research purposes by ISSDA. Sample sizes were *n* = 6400 for t_1_ and *n* = 5715 for t_2_, respectively.

### Outcome of interest: quality of life

QoL was assessed by means of the CASP-12 [12-item version of the CASP-19 by Hyde et al. [24]. Adopting a needs satisfaction perspective, the questionnaire was developed to assess need domains that explicitly represent positive and beneficial aspects of ageing. In this theoretical framework, QoL is defined as the satisfaction of these needs. On four-point items (from 0 “never” to 3 “often”), participants were asked to rate how often a statement described how they feel (e.g. “I feel that my life has meaning”). The original, 19-item version of the questionnaire consists of the four domains *self-realization, pleasure, control,* and *autonomy* [24]. For the present analysis, we used the abbreviated CASP-12 for the assessment of the two combined scores *self-realization/pleasure* (calculated from five items) and *control/autonomy* (calculated from seven items)*.* This two-factor model with the comprised subscales has been validated in the TILDA study previously and has shown superior fit to the four-subscale model in the sample [32]. According to Sexton and colleagues [32], the *control/autonomy* domain “(…) focuses on individual capacity to initiate and achieve valued goals, including the extent to which life circumstances (such as health, finances) restrict this ’’ (p. 2557). The *self-realization/pleasure* domain assesses “(…) the extent to which life is fulfilling, purposeful and involves connection to others” (ibid.). For the calculation of the sum scores, all items were coded so that higher scores represent higher QoL (possible ranges: *control/autonomy*: 0–21; *self-realization/pleasure*: 0–15).

### Main independent variables of interest

The presence of GAD and MD in the previous 12 months was assessed by means of the World Health Organization’s Composite International Diagnostic Interview—Short Form [CIDI-SF; 33] according to DSM-IV criteria. The CIDI-SF has been recommended for use in large, epidemiological studies and has shown a total classification accuracy of 99.6% for GAD and 93.2% for MD [33]. The CIDI-SF was conducted during the home assessment (CAPI) by trained interviewers.

### Other independent variables

For descriptive purposes, sex (male, female) and education (defined as the highest completed level of education, grouped into: “none or primary”, “secondary”, and “tertiary or higher”) were considered. Additional measures included in the analyses were age, marital status, retirement status, chronic conditions, cognitive function, and functional impairment.

Age was grouped into four categories (“50–59”, “60–69”, “70–79”, and “80 + ” years).

Marital status was grouped into “married”, and “not married”.

Retirement status included information on whether the participant was retired at the time of the assessment (yes, no).

Cognitive function was assessed by means of the Mini-Mental State Examination [MMSE; 34], using eleven items (e.g. assessment of orientation, attention, or recall), that are summed to calculate a total score (possible range 0–30). Higher scores indicate better cognitive function.

For the assessment of functional impairment, the participant was asked whether they were experiencing difficulties with five defined activities of daily living (ADLs; difficulty bathing or showering, dressing, eating [such as cutting up food], getting in or out of bed, walking across a room). The number of ADLs reported by the participant was categorized into four groups: “none”, “1 ADL”, “2–3 ADLs”, “4 + ADLs”.

Whether the participant was suffering from chronic or long-term health problems was assessed using a dichotomous variable (yes, no).

Lastly, to account for the receipt of mental health treatment, we included a variable assessing whether the participant reported (yes, no) the use of at least one antidepressant (ATC N06A).

### Variables for additional analysis

For an additional analysis, we included assessments on symptom severity of worry and depression instead of the CIDI-based categorical diagnoses of MD and GAD in the statistical analysis, which is described below in further detail.

The severity of worry was assessed by means of the abbreviated version [PSWQ-A; 35] of the Penn State Worry Questionnaire [36]. Eight items assess the severity of worry, the core symptom of GAD, and are rated on a five-point scale (from 1 “not at all typical” to 5 “very typical”). The answers are summed to a score reflecting the person’s severity of worry (possible range 8–40; higher scores indicate more severe worry). The PSWQ-A has shown adequate to good psychometric properties in older adults from clinical and non-clinical populations [37–39].

Depressive symptoms were assessed using a validated short-form of the Centre for Epidemiological Studies Depression Scale, which was developed for application in large-scale epidemiologic studies [40]. This screening instrument assesses the occurrence of depressive symptoms within the past week using eight items. Each item is rated on a four-point scale from 0 “rarely or none of the time (≤ 1 day)” to 3 “most of the time (5–7 days)”. A sum score can be calculated with higher scores representing more severe depressive symptoms (possible range 0–24). The CES-D-8 has shown good psychometric characteristics in the TILDA sample previously [41, 42].

### Statistical analysis

To investigate the association between specific transitions (incidence, remission) of GAD as well as MD and change in QoL over time, asymmetric linear fixed effects (FE) panel regression models were estimated. All adjusted FE regression models included as time-varying covariates age group, retirement status, marital status, presence of chronic conditions, number of difficulties in activities of daily living, cognitive function, and receipt of antidepressant medication. As two waves were used for the analysis, this corresponds to a first-difference approach. The major advantage of the FE approach is the possibility to control for time-constant unobserved heterogeneity. Unobserved heterogeneity is a critical problem in observational studies, because time-constant unobserved factors (e.g., genetic disposition) cannot be controlled in the analysis and could therefore bias the results [16]. However, even if time-constant unobserved factors are correlated with the regressors, FE regressions yield consistent estimates, because they exclusively rely on intrapersonal (or within) variance. Due to the focus on within variance, each person basically constitutes their own “control group” when calculating intrapersonal change between t_1_ and t_2_. Because (observed and unobserved) time-constant heterogeneity is eliminated from the models, the results should also only be generalized to those experiencing change over time, i.e. change in QoL for those transitioning into an episode of mental illness and vice versa. This interpretation of the coefficients resulting from FE models is conceptually similar to the average treatment effect on the treated [43].

In a symmetric linear FE model, we would assume that an increase or decrease of one unit of an independent variable would be interpreted as resulting in the increase or decrease in the outcome of the same magnitude. In our case, this would mean that the decrease in QoL associated with the incidence of a mental health disorder would be of the same magnitude as the increase in QoL associated with the remission of the respective disorder. In an asymmetric FE model [44], however, transitions over time are decomposed into their respective direction of change (e.g. difference scores are decomposed into increasing and decreasing symptom scores over time), and included as separate independent variables in the regression model. In this way, the effect of the different directions of transition can be determined separately, and possible asymmetry in this association can be assessed. To do the latter, Wald tests (*b*^+^  = − *b*^−^) were applied after the asymmetric FE model was calculated. This allowed us to check whether the estimates for increase in the independent variable (*b*^+^) were equal to the decrease in the independent variable (*b*^−^). If the null hypothesis of this post-estimation holds (*p* > 0.05), the estimates do not differ significantly and there is no sufficient evidence for asymmetry. The asymmetric FE approach for two-period data applied in this work, as well as its extensions, have been described in detail by Allison [44].

Because the FE regressions analyze intraindividual variability over time, they rely on the availability of data on the same individuals over time. Therefore, only people with available data on both waves for the variables of interest could be considered for the analysis. Missing values were not replaced. While we observed no or few missing values for most variables in our models, which were assessed during the personal CAPI in the participants’ homes, the proportion of missing values was larger for the SCQ, mostly because the SCQ was not returned for 15% of the participants at t1 and 14% at t2. This questionnaire was provided upon the completion of the interview together with a pre-paid envelop and included more sensitive topics (alcohol consumption, interpersonal relationships, and our main outcome QoL). In addition, not all participants took part in both waves. In sum, *n* = 5565 took part in both waves, out of which *n* = 4542 returned the SCQ (81.6%). The key findings reports for TILDA waves 3 [45]and 4 [46] suggest that those not returning the SCQ tended to be older with the lowest response rate in those aged 75 + . This resulted in a reduction of the sample size for our analysis, which relied on available data for our variables of interest at both waves and participants who also had changes in variables of interest over time. In sum, this resulted in sample sizes between *n* = 4284 and *n* = 3754 for unadjusted models. Missing values in covariates resulted in a further reduced sample size of n = 3995 and *n* = 3662 for the main covariate-adjusted models as well as *n* = 3668 and *n* = 3438 for the additional analyses.

In the additional analysis, we used the PSWQ-A and CES-D-8 scores instead of the CIDI-SF diagnoses to investigate whether using worry and depression symptom severity scores in the adjusted regression models, instead of the categorical diagnosis, would differ from the results observed in the main analysis.

The level of significance was set at *α* = 0.05. All statistical analyses were conducted with Stata 16 (Stata Corp, College Station, Texas).

## Results

### Descriptive sample characteristics

Detailed sample characteristics for participants are provided in Table 1. In short, at t_1_, over half of the participants were female (55.9%), 37.6% were between 60 and 69 years of age, and 39.8% had obtained a secondary level of education. Moreover, over half of the participants were not retired (53.6%) and were married (67.2%). About 57.5% reported no chronic conditions, and most participants reported no difficulties in ADLs (94.4%). At t_1_, the CASP-12 score means were *M* = 12.96 (SD: 2.49) for *self-realization/pleasure* and *M* = 14.01 (SD: 3.84) for *control/autonomy*, respectively.

**Table 1:** Descriptive information on waves 3 (t_1_) and 4 (t_2_) of the TILDA sample

Characteristic	t_1_ (wave 3, *n* = 6400)	t_2_ (wave 4, *n* = 5715)
Sex (*n*, %): male	2825 (44.1%)	2514 (44.0%)
Female	3575 (55.9%)	3201 (56.0%)
Age (*n*, %): 50–59 years	1746 (27.3%)	1092 (19.1%)
60–69 years	2406 (37.6%)	2298 (40.2%)
70–79 years	1544 (24.1%)	1578 (27.6%)
80 years and older	703 (11.0%)	747 (13.1%)
Highest level of education (*n*, %): none/primary	1623 (25.4%)	1344 (23.5%)
Secondary	2548 (39.8%)	2261 (39.6%)
Tertiary	2228 (34.8%)	2110 (36.9%)
Retired (*n*, %): yes	2907 (45.4%)	2790 (48.8%)
No	3430 (53.6%)	2898 (50.7%)
Marital Status (*n*, %): not married	2100 (32.8%)	1927 (33.7%)
Married	4300 (67.2%)	3788 (66.3%)
Presence of chronic conditions (*n*, %): no	3678 (57.5%)	3338 (58.4%)
Yes	2718 (42.5%)	2362 (41.3%)
Number of ADLs (*n*, %): 0	6043 (94.4%)	5323 (93.1%)
1	203 (3.2%)	255 (4.5%)
2–3	108 (1.7%)	96 (1.7%)
4 or more	37 (0.6%)	36 (0.6%)
MMSE (M, SD)	28.49 (2.06)	28.53 (2.13)
GAD (*n*, %): yes	210 (3.3%)	147 (2.6%)
No	5995 (93.7%)	5380 (94.1%)
MDE (*n*, %): yes	320 (5.0%)	261 (4.6%)
No	6069 (94.8%)	5446 (95.3%)
PSWQ-A (M, SD)	16.44 (8.13)	15.76 (7.95)
CES-D (M, SD)	3.26 (3.83)	3.23 (3.68)
Antidepressant medication (*n*, %): yes	592 (9.3%)	560 (9.8%)
No	5808 (90.8%)	5110 (89.4%)
CASP-self-realization/pleasure (M, SD)	12.96 (2.49)	13.18 (2.25)
CASP-control/autonomy (M, SD)	14.01 (3.84)	14.43 (3.82)

The prevalence of GAD was 3.3% at t_1_ and 2.6% at t_2_. Regarding transitions in GAD over time, we found that out of participants with data on GAD at both waves (*n* = 5,239), there were *n* = 89 (1.7%) with incident GAD (no GAD at t_1_, but GAD at t_2_) and *n* = 112 (2.1%) remitting cases (GAD at t_1_, but no GAD at t_2_). Regarding MD, the prevalence was 5.0% at t_1_ and 4.6% at t_2_. *N* = 5,551 participants provided data on MD at both waves. There were *n* = 182 incident cases (3.3%; no MD at t_1_, but MD at t_2_), and *n* = 206 remitting cases (3.7%; MD at t_1_, but no MD at t_2_) in the sample.

### Association between transitions in GAD and MD and change in QoL over time

Results on covariate-adjusted FE regressions (additionally adjusted for age groups, retirement status, marital status, presence of chronic conditions, number of ADLs, cognitive function, use of antidepressant medication) analyzing the association between transitions in GAD, MD and change in QoL over time are displayed in detail in Table 2. Results for covariates are displayed in the Appendix.

**Table 2:** Results from covariate-adjusted, asymmetric FE regressions analyzing the longitudinal association between transitions in GAD, MDE and change in QoL over time

Independent variables	Outcomes (change in CASP-12 domains)			
	Self-realization/pleasure		Control/autonomy	
	*b* (SE)	Wald test of coefficients	*b* (SE)	Wald test of coefficients
Incident GAD (ref: no incident GAD)	– 0.18 (0.28)	*F* (1, 3937) = 1.23, *p* = 0.267	– 0.34 (0.40)	*F* (1, 3644) = 0.20, *p* = 0.653
Remission of GAD (ref: no remission of GAD)	0.61* (0.26)		0.58 (0.36)	
Incident MDE (ref: no incident MDE)	– 0.08 (0.21)	*F* (1, 3937) = 3.22, *p* = 0.073	– 0.74* (0.30)	*F* (1, 3644) = 0.33, *p* = 0.567
Remission of MDE (ref: no remission of MDE)	0.61** (0.20)		0.50 (0.29)	
Covariates^a^	✔		✔	
Number of observations	3955		3662	
*R*^2^	0.012		0.009	

**p < 0.01, *p < 0.05

*CASP-12* 12-item version of the CASP-19 quality of life scale, *GAD* Generalized anxiety disorder, *MDE* Major depressive episode

Results obtained from asymmetric linear FE regressions (displayed as regression coefficients with standard errors in parentheses) and post-estimation Wald tests of the coefficients (*b*^+^  = − b^−^). Listwise deletion was used to handle missing values

^a^All models were additionally adjusted for age group, retirement status, marital status, presence of chronic conditions, number of difficulties in activities of daily living, cognitive function, and receipt of antidepressant medication (data for covariates shown in Online Appendix)

In short, incident GAD was not significantly associated with change in any QoL domain over time. Remission from GAD was significantly associated with gains in QoL over time in the *self-realization/pleasure* domain (*b* = 0.61, SE: 0.26, *p* = 0.018). The association between remission from GAD and change in *control/autonomy* scores over time was not significant.

Incident MD was significantly associated with a reduction in the *control/autonomy* score (*b* = − 0.74, SE: 0.30, *p* = 0.013) but not the *self-realization/pleasure* domain. Remission from MD was significantly associated with improvement in QoL over time in the *self-realization/pleasure* domain (*b* = 0.61, SE: 0.20, *p* = 0.003). The association between remission from MD and change in *control/autonomy* scores over time was not significant.

Post-estimation Wald tests examining the equality of the coefficients (*b*^GAD incidence^ = − *b*^GAD remission^; *b*^MD incidence^ = − *b*^MD remission^) in all models were not significant, indicating symmetric effects.

### Additional analysis: applying symptom scores instead of categorical diagnoses

In the additional analysis, we included the PSWQ-A and CES-D-8 symptom scores in the covariate-adjusted model (adjusted for age groups, retirement status, marital status, presence of chronic conditions, number of ADLs, cognitive function, use of antidepressant medication) instead of the CIDI-SF diagnoses, to check whether the result observed in the main analysis would remain. Results for the symptom scores are displayed in Table 3 and results for covariates in further detail in the Appendix.

**Table 3:** Results from additional analyses including transitions in PSWQ-A and CES-D-8 and change in QoL scores over time

Independent variables	Outcomes (change in CASP-12 domains)			
	Self-realization/pleasure		Control/Autonomy	
	*b* (SE)	Wald test of coefficients	*b* (SE)	Wald test of coefficients
PSWQ-A increase (ref: no increase)	– 0.04*** (0.01)	*F* (1, 3650) = 0.02, *p* = 0.876	– 0.10*** (0.02)	*F* (1, 3420) = 0.96, *p* = 0.328
PSWQ-A decrease (ref: no decrease)	0.05*** (0.01)		0.08*** (0.01)	
CES-D-8 increase (ref: no increase)	– 0.03 (0.02)	*F* (1, 3650) = 3.33, p = 0.068	– 0.03 (0.02)	*F* (1, 3420) = 0.74, *p* = 0.390
CES-D-8 decrease (ref: no decrease)	0.08*** (0.02)		0.06** (0.02)	
Covariates^a^	✔		✔	
Number of observations	3668		3438	
*R*^2^	0.032		0.038	

*CASP-12* 12-item version of the CASP-19 quality of life scale, *PSWQ-A* abbreviated version of the Penn State Worry Questionnaire, *CES-D-8* eight-item Center for Epidemiological Studies Depression Scale

***p < 0.001, **p < 0.01

Results obtained from asymmetric linear FE regressions (displayed as regression coefficients with standard errors in parentheses) and post-estimation Wald tests of the coefficients (*b*^+^  = − b^−^). Listwise deletion was used to handle missing values

^a^All models were additionally adjusted for age group, retirement status, marital status, presence of chronic conditions, number of difficulties in activities of daily living, cognitive function, and receipt of antidepressant medication (data for covariates shown in Online Appendix)

In short, regarding PSWQ-A scores, we found that increasing worry levels over time were significantly associated with reduced QoL in both domains, and reduced worry levels over time were associated with an increase in both QoL domains.

Regarding the CES-D-8 scores, increasing depressive symptom levels over time were not significantly associated with a reduction in QoL over time. Reduced depressive symptom levels over time were significantly associated with increases in both QoL scores.

As in the main analysis, post-estimation Wald tests of the symptom score trajectory coefficients (*b*^PSWQ-A increase^ = − *b*^PSWQ-A decrease^; *b*^CES−D-8 increase^ = − *b*^CES−D-8 decrease^) were not significant, indicating symmetric effects.

## Discussion

The aims of this study were: first, to investigate the longitudinal association between transitions in GAD and MD and change in domain-specific QoL in a population-based sample of adults aged 50 +  as well as second, to assess the symmetry of the observed relationship.

Regarding the first aim of this study, in the main analysis we found that remission of both GAD and MD was associated with significant improvements in QoL over time (*self-realization/pleasure* domain). Incident MD but not GAD was significantly associated with a reduction in QoL over time (*control/autonomy* domain).

Generally, our results concur with a previous longitudinal study from the general population showing a significant association between the remission of GAD or MD and increases in QoL [12]. However, previously a significant association between new onset GAD as well as MD and reduction in QoL was observed in the general population [11]. Interestingly, while incident GAD was not significantly associated with reduced QoL over time, an increase in dimensional worry, the core symptom of GAD, was significantly associated with reduction in both QoL domains. This points to the relevance of dimensional anxiety symptoms when analyzing QoL outcomes in older adults, besides the dichotomous diagnostic category. Reasons for different findings could also be due to differences in the samples or QoL domains analyzed as well as in the method of analysis. More specifically, Rubio et al. [11] analyzed data from the NESARC study, which also included younger people (people aged 18 + were included) and focused on mental component summary scores of the SF-12. Regarding the method of analysis, the study applied linear regressions comparing QoL changes in people with incident GAD to people without a lifetime history of the disorder or any psychiatric disorder. As described in detail in the methods section, in the FE regressions applied in our analysis, the focus is a within-variation analysis.

Comparing our domain-specific results with previous studies is challenging because this is, to our knowledge, the first study using longitudinal data to investigate GAD and MD trajectories and their association with change in CASP-12 subscales. Previous evidence from systematic reviews has found that mental disorders may be associated differently with individual QoL domains in longitudinal [25] and cross-sectional studies [47]. In addition, several cross-sectional analyses of older people have reported on the association between CASP total scores and depression measures only [48–52] as well as in association with depression and anxiety measures [53]. To our knowledge, only a few previous studies in older adults have investigated depression or depressive symptoms and CASP scores longitudinally [20–23]. For example, in their longitudinal analyses based on the ELSA sample, Webb et al. [20] and de la Torre-Luque et al. [23] applied the CASP-19 total score. They found that becoming depressed and recovering from depression according to CES-D cutoffs were among the strongest predictors of change in the QoL measure [20], and that showing subclinical and chronic depression symptom trajectories resulted in lower CASP-19 scores at follow-up compared to the normative trajectory category [23]. Similarly, Ward et al. [21] analyzed CASP-12 total scores in the TILDA sample and found a negative association between depressive symptoms and QoL scores over a 4-year period.

Our study expands the findings from the previous longitudinal, population-based studies by focusing on individual QoL domains, by considering anxiety, and by using mental health diagnoses based on a structured clinical interview. In our study, there was a significant association between GAD and MD diagnoses and the domain *self-realization/pleasure* of the CASP-12. Improvements in this domain due to the remission of GAD and MD may reflect that those experiencing a significant reduction of symptoms, resulting in them not fulfilling the criteria for these disorders anymore, tend to increase the active pursue of activities that they see as fulfilling and bring them joy as well as connection to others, which is particularly represented in this domain [24, 32]. On the level of remission of the clinical disorders, the coefficients for *control/autonomy* showed the expected increase in QoL levels which was, however, not significant. Interestingly, on the level of symptom severity, we observed a significant association between reduction of worry and depression symptoms with increased scores in *both* domains. This shows that an improvement in mental health symptoms was beneficial for the affected individuals on the level of QoL as well. That may particularly be the case for adults aged 50 + who do not fulfill the diagnostic criteria for the mental disorders, but still feel impacted by worry or depression symptoms.

While the results regarding the first aim of this study may hint to some trajectory-specific differences in the association between GAD, MD and QoL in adults aged 50 + , and therefore possibly asymmetric effects, the statistical analyses of the symmetry of the estimates (second aim) rather found evidence for a symmetric relationship. This indicates that a reduction in QoL associated with incidence of the disorders was of similar magnitude as the increases in QoL associated with remission of the disorders in this sample.

There is only little previous observational, population-based research on symmetry or asymmetry in QoL change scores associated with MD and GAD, which makes comparisons challenging. Existing studies were based on *three* waves of a population-based cohort study and explicitly examined scarring effects. They concluded that pre- and post-morbid QoL levels did not differ significantly within the same individuals experiencing incidence and remission of MD and anxiety disorders [17, 18]. In contrast, our analysis was based on *two* waves of the TILDA cohort. Therefore, we investigated change in QoL levels by means of FE regressions in two groups of people of the same sample: one group experiencing incident GAD or MD, and another group experiencing remission in the same time frame, and found that those QoL changes appear to be of similar magnitude in adults aged 50 + using post-estimation Wald tests. Based on our analysis of different groups experiencing incidence or remission of the disorders during the same timeframe, we cannot definitively conclude that there is no scarring effect. The evidence for a symmetric association in our study of older adults and the observation of mostly no scarring effects in mixed-age samples [17, 18] could, nonetheless, be interpreted as a positive finding for those suffering from MD or anxiety disorders. These findings indicate that QoL lost due to the disorder can be regained after remission, and that residual impairments in QoL should be small. In our study, this was even found at the symptom level. Yet, it should be noted that population-based studies also report a lower level of QoL in people with MD or anxiety disorders compared to healthy controls, and even lower levels for people experiencing a more severe illness episode [17, 18]. Regarding implications, this indicates a significant vulnerability and need for adequate support in these groups that should be analyzed in future studies of older adults. Future longitudinal studies in older adults should build upon this work and focus on different QoL domains and disorder trajectories, as these allow for more detailed and nuanced conclusions in this complex association, as discussed in detail in a recent review and meta-analysis of the association between anxiety, depression and QoL based on general population data [25]. Moreover, regarding clinical implications, specifically addressing and monitoring QoL domains as well as dimensional symptom profiles besides focusing on disorder incidence or remission may be beneficial for prevention and treatment to be able to identify and target specific areas where additional support may be needed more specifically in adults aged 50 + .

### Strengths and limitations

A certain strength of this work is the data that was used for the analyses. The TILDA study is a large, representative sample of adults aged 50 and over in Ireland and the sample was drawn by means of a comprehensive, complex sampling strategy. Another strength is the measures that were used for the operationalization of the main variables. For example, Sivertsen et al. [54] have identified a lack of theory-based QoL assessments, as well as seldom use of structured interviews for the assessment of depression, in previous literature regarding QoL in older age. In the present analysis, we used a theory-based QoL assessment that was specifically developed for older adults as well as a structured clinical interview (CIDI-SF) for the assessment of mental disorders.

A limitation is that, due to the sampling procedure focusing on people aged 50 + with residential addresses, nursing home residents and people residing in other institutions were not included at baseline. Therefore, our results may at first be generalized to adults aged 50 + living in the community, rather than possibly frailer or sicker people residing in institutions. Nevertheless, we used data from the last two waves of the TILDA study. It is to be expected that, over time, some participants that were not in a nursing home at baseline were residing in one during follow-up. A supposed drawback of the applied statistical method is that FE does not allow to estimate the effect of time-constant factors in this association, e.g. persistent mental health disorder or other factors that are usually time-constant in older age, such as education. However, the estimation of time-constant factors was not the primary aim of this work. Moreover, and more importantly, it has to be noted that the FE approach offers the major advantage of reducing unobserved heterogeneity bias, which is a critical problem in observational studies where unobserved, time-constant confounders are usually not accounted for and can bias the estimates [55]. Yet, while longitudinal data lend themselves relatively better to causal arguments compared to cross-sectional analyses, longitudinal observational data, even with application of FE, cannot provide definitive proof of a causal relationship. For example, the possibility of a bidirectional relationship or reverse causality, i.e. change in QoL could influence subsequent change in mental health, has to be considered. This has been demonstrated previously in older adults [56]. Moreover, as described by Carey [45] the most frequent reasons for attrition at wave 4 were refusals (for reasons of time constraints) as well as permanent withdrawal from the study. Thus, there is the possibility of some attrition bias. However, it should be noted that attrition associated with person-specific characteristics (both, observed and unobserved) do not bias the estimates of linear FE regressions [43].

## Supplementary Information

Below is the link to the electronic supplementary material.

Supplementary file1 (DOCX 21 KB)

## Data Availability

This is a secondary analysis of wave 3 and wave 4 data from The Irish Longitudinal study on Ageing (TILDA): The Irish Longitudinal study on Ageing (TILDA) Wave 3 (2014–2015), Wave 4 (2016). Accessed via the Irish Social Science Data Archive—www.ucd.ie/issda.
