# Supplementary material for: Trajectories of generalized anxiety disorder, major depression and change in quality of life in adults aged 50 + : findings from a longitudinal analysis using representative, population-based data from Ireland
Source: Soc Psychiatry Psychiatr Epidemiol. 2022 Oct 13;58(8):1201–11. doi: 10.1007/s00127-022-02373-0 (PMC10366232; doi:10.1007/s00127-022-02373-0)
Supplement: Supplementary file 1 — Supplementary file1 (DOCX 21 KB) [file 127_2022_2373_MOESM1_ESM.docx]

**Appendix**

**Table A1. Results from asymmetric FE regressions analyzing change in QoL outcomes over time (main analysis)**

|  | **Outcomes (change in CASP-12 domains)** | |
| --- | --- | --- |
| **Independent variables** | *self-realization/pleasure* | *control/autonomy* |
| incident GAD | -0.18 (0.28) | -0.34 (0.40) |
| remission of GAD | 0.61* (0.26) | 0.58 (0.36) |
| incident MDE | -0.08 (0.21) | -0.74* (0.30) |
| Remission of MDE | 0.61** (0.20) | 0.50 (0.29) |
| Increasing age | 0.16 (0.08) | 0.08 (0.12) |
| Transitioning into retirement | 0.08 (0.11) | -0.02 (0.16) |
| Transitioning out of retirement | -0.38* (0.15) | -0.36 (0.22) |
| Transitioning into marriage | -0.68 (0.51) | -1.60* (0.69) |
| Transitioning out of marriage | -0.50 (0.29) | 0.12 (0.43) |
| Increase in number of chronic conditions | 0.03 (0.11) | -0.24 (0.15) |
| Reduction in number of chronic conditions | 0.03 (0.10) | 0.06 (0.15) |
| Increase in activities of daily living | -0.16 (0.13) | -0.08 (0.19) |
| Reduction in activities of daily living | 0.18 (0.17) | -0.06 (0.24) |
| Increase in cognitive function | 0.01 (0.04) | -0.04 (0.06) |
| Reduction in cognitive function | -0.06* (0.03) | -0.09* (0.04) |
| Start use of antidepressant medication | -0.22 (0.21) | 0.24 (0.30) |
| Stop use of antidepressant medication | 0.28 (0.26) | 0.50 (0.37) |
| Constant | 0.20*** (0.05) | 0.40*** (0.07) |
| Observations | 3,955 | 3,662 |
| R^2^ | 0.012 | 0.009 |

**Table A2. Results from additional analyses (applying symptom severity scores instead of categorical diagnoses in asymmetric FE regressions analyzing change in QoL outcomes over time)**

|  | **Outcomes (change in CASP-12 domains)** | | | | |
| --- | --- | --- | --- | --- | --- |
| **Independent variables** | *Self-realization/pleasure* | | | *control/autonomy* | |
| Increase in PSWQ-A scores | | -0.04*** (0.01) | -0.10*** (0.02) | |  |
| Reduction in PSWQ-A scores | | 0.05*** (0.01) | 0.08*** (0.01) | |  |
| Increase in CES-D-8 scores | | -0.03 (0.02) | -0.03 (0.02) | |  |
| Reduction in CES-D-8 scores | | 0.08*** (0.02) | 0.06** (0.02) | |  |
| Increasing age | | 0.15 (0.08) | -0.003 (0.12) | |  |
| Transitioning into retirement | | 0.04 (0.12) | 0.03 (0.17) | |  |
| Transitioning out of retirement | | -0.36* (0.16) | -0.40 (0.23) | |  |
| Transitioning into marriage | | -0.81 (0.48) | -1.86** (0.66) | |  |
| Transitioning out of marriage | | -0.56 (0.30) | 0.13 (0.43) | |  |
| Increase in number of chronic conditions | | -0.01 (0.11) | -0.24 (0.15) | |  |
| Reduction in number of chronic conditions | | 0.07 (0.10) | 0.17 (0.15) | |  |
| Increase in activities of daily living | | -0.18 (0.13) | -0.01 (0.19) | |  |
| Reduction in activities of daily living | | -0.18 (0.17) | -0.09 (0.24) | |  |
| Increase in cognitive function | | -0.04 (0.04) | -0.05 (0.06) | |  |
| Reduction in cognitive function | | -0.06* (0.03) | -0.12** (0.04) | |  |
| Start use of antidepressant medication | | -0.17 (0.21) | 0.28 (0.30) | |  |
| Stop use of antidepressant medication | | 0.30 (0.27) | 0.37 (0.39) | |  |
| Constant | | 0.16** (0.06) | 0.38*** (0.09) | |  |
| Observations | | 3,668 | 3,438 | |  |
| R^2^ | | 0.032 | 0.038 | |  |
